# Metabolic regulation of the PMCA: Role in cell death and survival^☆^

**DOI:** 10.1016/j.ceca.2017.06.001

**Published:** 2018-01

**Authors:** Jason I.E. Bruce

**Affiliations:** Division of Molecular & Clinical Cancer Sciences, School of Medical Sciences, Faculty of Biology, Medicine and Health, University of Manchester, United Kingdom

**Keywords:** PMCA, Calcium pump, Calcium overload, Necrosis, Apoptosis, ATP

## Abstract

The plasma membrane Ca^2+^-ATPase (PMCA) is a ubiquitously expressed, ATP-driven Ca^2+^ pump that is critical for maintaining low resting cytosolic Ca^2+^ ([Ca^2+^]_i_) in all eukaryotic cells. Since cytotoxic Ca^2+^ overload has such a central role in cell death, the PMCA represents an essential “linchpin” for the delicate balance between cell survival and cell death. In general, impaired PMCA activity and reduced PMCA expression leads to cytotoxic Ca^2+^ overload and Ca^2+^ dependent cell death, both apoptosis and necrosis, whereas maintenance of PMCA activity or PMCA overexpression is generally accepted as being cytoprotective. However, the PMCA has a paradoxical role in cell death depending on the cell type and cellular context. The PMCA can be differentially regulated by Ca^2+^-dependent proteolysis, can be maintained by a localised glycolytic ATP supply, even in the face of global ATP depletion, and can be profoundly affected by the specific phospholipid environment that it sits within the membrane. The major focus of this review is to highlight some of the controversies surrounding the paradoxical role of the PMCA in cell death and survival, challenging the conventional view of ATP-dependent regulation of the PMCA and how this might influence cell fate.

## Highlights

• The PMCA is an ATP-driven Ca^2+^ pump critical for the maintenance of low cytosolic calcium.
• The PMCA has an important but paradoxical role in cell death and survival.
• The PMCA can be differentially regulated by caspase/calpain cleavage.
• Glycolytic ATP supply may be sufficient to fuel the PMCA during metabolic stress.
• The ATP sensitivity of the PMCA can be regulated by acidic phospholipids.

## Graphical abstract

**Figure:**
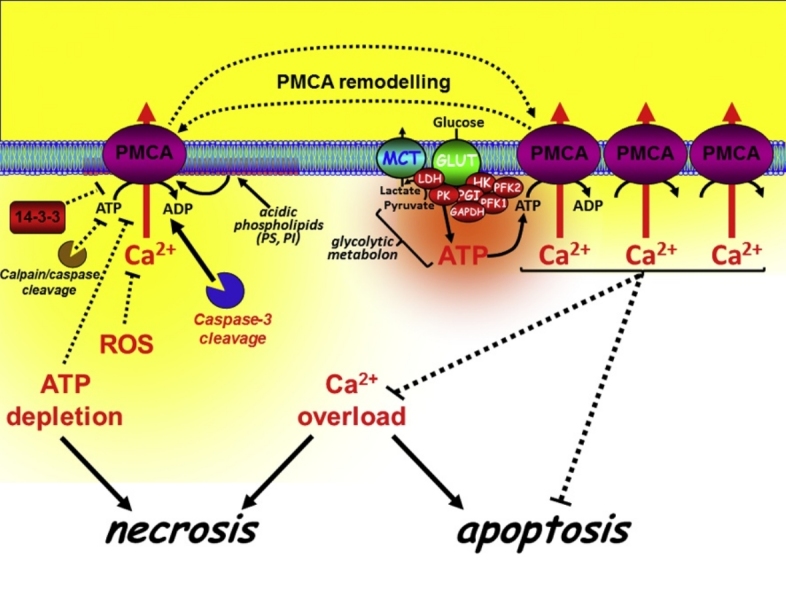

## 1 Introduction

The plasma membrane Ca^2+^-ATPase (PMCA) is an ATP-driven Ca^2+^ pump ubiquitously expressed in the plasma membrane of all eukaryotic cells. PMCA is encoded by four separate genes (PMCA1-4) and numerous splice variants that give rise to specific tissue distribution, cellular localisation and functional diversity [1], [2]. PMCA1 and PMCA4 are ubiquitously expressed whereas PMCA2 and PMCA3 have a more tissue specific expression, mainly in excitable cells [3]. The PMCA is critical for maintaining cytosolic Ca^2+^ concentration ([Ca^2+^]_i_) below 300 nM (∼100 nM), due to its high affinity for Ca^2+^ (Kd, ∼0.2 μM) [4], [5], [6] and is the major Ca^2+^ efflux pathway in non-excitable cells [7]. For many years the PMCA was thought to have a “minor” house-keeping role in maintaining low resting [Ca^2+^]_i_ [8]. However, the importance of PMCA in the spatiotemporal shaping of cytosolic Ca^2+^ signalling has steadily increased. PMCA exhibits memory of past [Ca^2+^]_i_ increases, suggesting an important role in regulating the frequency of Ca^2+^ oscillations [9]. Moreover, the different PMCA isoforms, and numerous splice variants of PMCA, can be differentially expressed in specific regions of cells and can also be differentially regulated by a sophisticated repertoire of additional signalling pathways [10], [11], [12], [13].

Despite the emerging role of the PMCA in dynamic Ca^2+^ signalling, the importance of the house-keeping role of the PMCA should not be under-estimated, especially when one considers how important maintaining low resting [Ca^2+^]_i_ is for cell survival and the prevention of Ca^2+^-dependent cell death. In this regard the PMCA can be regarded as the “last gatekeeper” for the maintenance of low resting [Ca^2+^]_i_; an essential “linchpin” for the delicate balance between cell survival and cell death [14], [15], [16], [17]. Moreover, the PMCA is inextricably linked to the specific nature of cell death. Not only does PMCA prevent Ca^2+^ overload associated apoptosis, but the PMCA is an ATP-driven pump and since ATP depletion induces necrosis, a decline in PMCA activity will accompany and exacerbate necrosis [17], [18], [19], [20]. Therefore, PMCA activity may act as an important switch between apoptosis and necrotic cell death, a key determinant of numerous disease processes. Thus the maintenance of PMCA activity is critical for cell survival, particularly in the face of modest-to-severe global ATP depletion, whereas inhibition of PMCA even when global ATP is maintained will facilitate Ca^2+^-dependent apoptosis.

## 2 Physiological regulation and key structural features of the PMCA

**Fig. 1:**
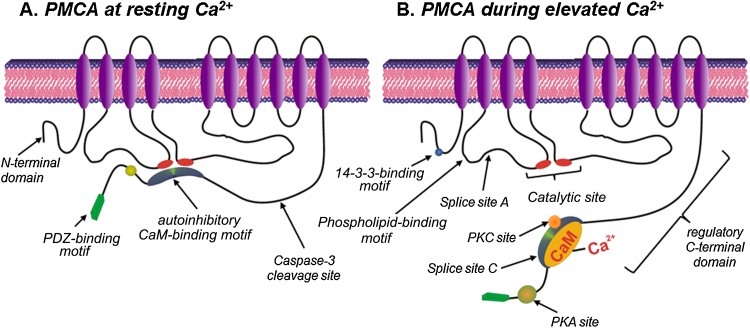
Two-dimensional topological model of the structure of the PMCA at low resting [Ca^2+^]_i_ and following activation at elevated [Ca^2+^]_i_. ***A***., at low resting [Ca^2+^]_i_ the autoinhibitory CaM binding motif within the C-terminal tail of the PMCA associates with the catalytic motif, thereby preventing Ca^2+^ binding and thus Ca^2+^ transport. ***B.***, when [Ca^2+^]_i_ is elevated, Ca^2+^/CaM binds to the autoinhibitory CaM binding motif inducing a conformational change that causes dissociation from the catalytic motif, thereby allowing access to Ca^2+^ and thus the transport of Ca^2+^. Additional regulatory motifs include an inhibitory 14-3-3-binding site in the N-terminal region, a stimulatory phospholipid-binding motif in the first cytosolic loop and PKA/PKC phosphorylation consensus sites and a PDZ binding motif in the C-terminal tail. Splice sites A and C can generate splice variants with specific tissue-specific distribution, cellular localization and differential regulation.

In order to understand the role of the PMCA in cell death and survival it is necessary to highlight some of the key structural features and regulatory mechanisms. Structurally, PMCA consists of ten transmembrane domains, two cytosolic loops with both N- and C-terminal cytosolic tails (Fig. 1) [1], [2]. Arguably the most functionally important structural domain is the C-terminal tail which contains the autoinhibitory calmodulin (CaM)-binding motif [21]. At low resting [Ca^2+^]_i_, the autoinhibitory CaM-binding motif interacts with the catalytic site (first and second cytosolic loops) thereby inhibiting the PMCA (Fig. 1A). When [Ca^2+^]_i_ is elevated, binding of Ca^2+^/CaM to this autoinhibitory motif induces a conformational change which reduces its affinity for the catalytic site thereby increasing the Ca^2+^ transporting activity of the PMCA (Fig. 1B) [5]. The C-terminus also contains additional high affinity allosteric Ca^2+^ binding sites [22] and an acidic phospholipid binding site [23], [24]. Binding of acidic phospholipids such as phosphatidylinositol (PI) and phosphatidylserine (PS) increases the Ca^2+^ and ATP affinity of the PMCA. Phosphoinositide 4,5-bisphosphate (PIP_2_) is also a major activator of PMCA and is thought to account for ∼50% of the activity of PMCA at rest [4], [5]. The last few amino acid residues of the C-terminus of the PMCA contain PDZ-binding motif, which facilitate PMCA dimerization [25] which increases PMCA activity [26]. In addition, the PDZ-binding motif also facilitates the recruitment of the actin cytoskeleton [27], numerous scaffolding proteins and signalling complexes [28], [29], [30], [31], [32], [33]. Such targeting only occurs for the full-length b-variants, suggesting specialised signalling roles for different PMCA isoforms. Specifically, PMCA4b functionally interacts with neuronal nitric oxide synthase (nNOS) [34], [35], calcineurin [36] and the pro-apoptotic tumour suppressor Ras-associated factor 1 (RASSF1) [37], thereby regulating their downstream signalling.

The N-terminal tail exhibits the greatest diversity between the different isoforms [38] and contains an inhibitory 14-3-3-binding motif [39], [40]. In addition to part of the binding site for the autoinhibitory calmodulin (CaM)-binding motif [41] (see Fig. 1B), the first cytosolic loop of the PMCA, which spans between the second and third transmembrane domains, contains a stimulatory acidic phospholipid-binding site [42], [43] and splice site A important for the apical membrane targeting in epithelial cells [44], [45], [46]. The second cytosolic loop between the fourth and fifth transmembrane domains contains the major catalytic site (including the critical aspartate residue that becomes phosphorylated during the reaction cycle and ATP binding) and the second part of the binding site for the autoinhibitory CaM-binding motif within the C-terminal tail [47].

## 3 The controversial role of PMCA in cell death

### 3.1 Intrinsic Ca^2+^-dependent cell death

**Fig. 2:**
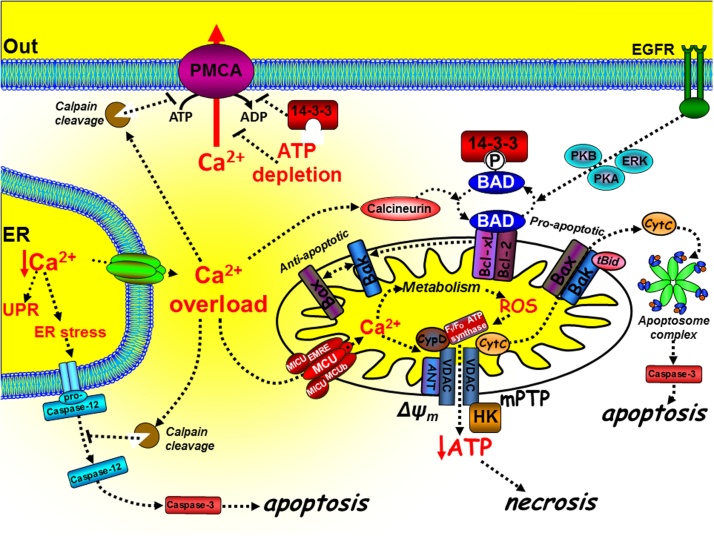
The mechanisms of intrinsic Ca^2+^-dependent cell death. Cytotoxic Ca^2+^ overload can mediate intrinsic cell death at both the mitochondria and ER. At the mitochondria, tBid binds to and promotes Bax and Bak oligomerisation [50] which form pores through which cytochrome C can be released into the cytosol. Cytochrome C binds to and forms part of the apoptosome complex which activates the executioner caspases, such as caspase-3, leading to the “point-of-no-return” apoptotic cascade [48], [51]. The anti-apoptotic proteins, Bcl-2 and Bcl-xL, can prevent the t-Bid/Bax/Bak interaction, thereby preventing apoptosis. The pro-apoptotic protein Bad binds to Bcl-2/Bcl-xL [53], [129], thereby preventing their interaction with the t-Bid/Bax/Bak complex and thus promoting apoptosis [130]. Phosphorylation of Bad by the protein kinases, PKB [131], PKA [132] and ERK [133], causes Bad to dissociate from the mitochondria and bind to 14-3-3 protein. Sustained Ca^2+^ overload can activate calcineurin which dephosphorylates Bad allowing it to sequester the anti-apoptotic proteins, Bcl-2/Bcl-xL [54]. Ca^2+^ uptake into the mitochondria, via the mitochondrial Ca^2+^ uniporter (MCU), and its associated proteins (MICU, MCUb, EMRE), can lead to the production of reactive oxygen species (ROS). Ca^2+^ and ROS can activate the permeability transition pore (mPTP), loss of the mitochondrial membrane potential (ΔYm), ATP depletion and necrosis. Ca^2+^ overload can also activate calpain and cleavage of the PMCA.

Since the PMCA is critical for the regulation of low resting [Ca^2+^]_i_ and the prevention of cytotoxic Ca^2+^ overload, in order to understand the role of PMCA in cell death and survival it is important to explore the mechanisms of Ca^2+^-dependent cell death. Ca^2+^ has a critical yet paradoxical role in regulating cell death [48] and both ER Ca^2+^ and mitochondrial Ca^2+^ are key contributors (Fig. 2). Cells adopt strategies to avoid cell death by activating pro-survival pathways and suppressing cell death machinery, of which the main players include the pro- and anti-apoptotic B-cell lymphoma 2 (Bcl-2) family of proteins of which there are around 20 different members [49]. At the mitochondria, tBid binds to and promotes Bax and Bak oligomerisation [50] which form pores through which cytochrome C can be released into the cytosol. Cytochrome C binds to and forms part of the apoptosome complex which activates the executioner caspases, such as caspase-3, leading to the “point-of-no-return” apoptotic cascade [48], [51]. The balance between pro-apoptotic (Bax, Bak and Bad) and anti-apoptotic proteins (Bcl-2/Bcl-xL) determines whether a cell is sensitive or resistant to apoptosis [52] (Fig. 2). The phosphorylation status of Bad is a critical checkpoint for apoptosis (Fig. 2). When phosphorylated by protein kinase-A (PKA), protein kinase-B (PKB) or Ras-mitogen activated kinase (MAPK) Bad dissociates from the mitochondria to the cytosol where it binds to 14-3-3 protein. This prevents Bad from engaging and inhibiting Bcl-2/Bcl-xL, thereby inhibiting apoptosis [53] (Fig. 2). Moreover, the Ca^2+^-dependent activation of calcineurin leads to the dephosphorylation of Bad, allowing it to bind to and inhibit Bcl-2/Bcl-xL and thus promote apoptosis [54]. Of relevance to the PMCA, 14-3-3 proteins directly bind to and inhibit the PMCA [39], thereby accentuating the Ca^2+^/calcineurin-mediated dephosphorylation of Bad and further potentiating this Ca^2+^-mediated apoptosis.

**Fig. 3:**
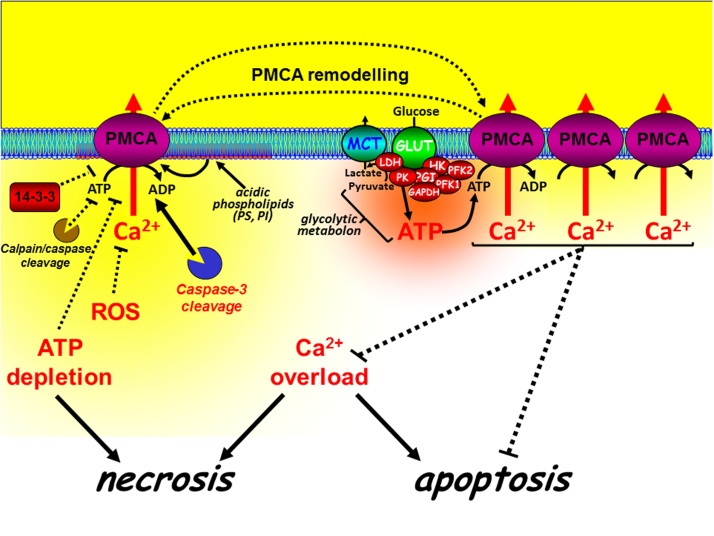
Differential regulation and paradoxical role of PMCA in cell death. The PMCA is a high affinity Ca^2+^ clearance pathway critical for maintaining low resting [Ca^2+^]_i_ and thus prevention of cytotoxic Ca overload-induced cell death (both necrosis and apoptosis). Inhibition or reduced expression of the PMCA generally increases cell death, whereas activation or overexpression of the PMCA generally inhibits cell death. The PMCA can be directly inhibited by 14-3-3 binding, calpain/caspase cleavage, oxidation by reactive oxygen species (ROS) or by ATP depletion during metabolic stress. However, specific caspase-3 cleavage of the PMCA can remove the autoinihitory CaM-binding domain, resulting in a truncated constitutively active PMCA. Moreover, acidic phospholipids, such as phosphatidylserine (PS) and phosphatidylinositol (PI) can markedly increase the affinity of the PMCA for ATP and Ca^2+^/CaM, making the PMCA largely insensitive to moderate ATP depletion. Furthermore, glycolytic enzymes can associate with the plasma membrane to form a metabolic complex (metabolon) which provides the PMCA with a privileged ATP supply to the PMCA, even during global ATP depletion. These mechanisms may be important for maintaining survival during severe metabolic stress.

Ca^2+^ has a more prominent role during cell stress-induced cell death [48] (Fig. 3). Mitochondria take up Ca^2+^, via the mitochondrial Ca^2+^ uniporter (MCU) [55], [56], which was originally believed to have a low affinity for Ca^2+^ and thus facilitate Ca^2+^ uptake into the mitochondrial matrix only when cytosolic Ca^2+^ is very high. However, the molecular identity of MCU, and its various pore-forming subunits (MCUb and “essential MCU regulator” (EMRE)) and accessory proteins (MICU1 and MICU2), has revealed that this process is a complex molecular machine [57]. Depending on the relative expression of each of these molecular components and cellular context this process exhibits a more complex gating and Ca^2+^-dependency than originally thought [57]. Nevertheless, MCU can facilitate the physiological Ca^2+^ dependent activation of key metabolic enzymes [58], in a process referred to as stimulus-metabolism coupling. Alternatively during excessive cytosolic Ca^2+^ overload, which can occur when the PMCA is inhibited, MCU can lead to excessive mitochondrial Ca^2+^ overload and the production of reactive oxygen species (ROS) [59]. Both ROS and Ca^2+^ can activate the mitochondrial permeability transition pore (mPTP) [59], a molecular machine which consists a growing list of regulatory and accessory proteins, but with core members including the voltage-dependent anion channel (VDAC), adenine nucleotide translocase (ANT) and cyclophilin-D [60] (Fig. 3). The molecular composition and regulatory mechanism of the mPTP is hotly debated and a fast growing field and thus beyond the scope of this review. However, in simple terms the mPTP is responsible for coupling mitochondrial volume and ion homeostasis with metabolism and cellular stress. Excessive mPTP activation leads to mitochondrial swelling, outer mitochondrial membrane rupture and cytochrome C release [48]. For many years the mPTP was thought to be important for Ca^2+^-mediated apoptosis [61], [62], however knockout studies of VDAC [63] and cyclophilin-D [64], [65] had no effect on apoptosis but attenuated necrotic cell death. This suggests that the mPTP may be more important during necrotic cell death than for apoptosis. Nevertheless, it is clear that the mPTP can functionally interact with Bcl-2/Bcl-xL and Bax/Bak [61], [62], [66], [67], suggesting that necrosis and apoptosis may share some of the same molecular machinery. The defining feature of necrosis is that this is usually accompanied by the collapse of the mitochondrial membrane potential, due to excessive activation of the mPTP and the consequent inhibition of mitochondrial ATP synthesis. This can have a knock-on effect on the ATP-dependent PMCA, as well as SERCA and the Na^+^/K^+^-ATPase.

Ca^2+^ can also amplify cell death by activating the proteolytic enzymes, calpains [68], which can directly activate caspases [69] and can inactivate the anti-apoptotic Bcl-2 [70] and the PMCA [20], [71], [72], [73], [74] (Fig. 2). Paradoxically, some of the pro-apoptotic and anti-apoptotic proteins have been reported to directly regulate key Ca^2+^ transport proteins [75], [76], [77], including the PMCA [78] resulting in a complex reciprocal regulation between Ca^2+^ signalling and cell death.

Enhanced Ca^2+^ signalling and cytosolic Ca^2+^ overload clearly promotes both apoptosis and necrosis. Intuitively therefore, impaired PMCA activity or reduced PMCA expression would be expected to promote Ca^2+^-mediated cell death, whereas maintenance of PMCA activity or PMCA overexpression is generally regarded to be cytoprotective. Although such remodelling of PMCA function and expression may have evolved to attenuate Ca^2+^-mediated cell death, paradoxically, this may also result in apoptosis resistance which is a major hallmark of cancer.

### 3.2 The PMCA paradox

Although the physiological role of the PMCA has been debated for several years, the importance of the PMCA during cellular stress and under pathological conditions is undeniable. The nature of cell death (i.e. necrosis vs apoptosis) will largely depend on the extent of metabolic stress and cytosolic Ca^2+^ overload. However, ATP depletion during extensive metabolic stress has been suggested to be the determining factor whether a cell undergoes apoptosis or necrosis [79], [80], [81], regardless of whether this is accompanied by Ca^2+^ overload. This is largely because ATP is critical for many of the ATP-dependent apoptotic processes, but is not required for necrosis [81]. During pancreatitis, apoptosis is generally regarded as protective, as this involves the safe dismantling of the cell constituents [82], [83]. Necrosis, on the other hand, is an uncontrolled form of cell death characterised by cell lysis, and the subsequent release of activated proteases (zymogens) from the effected pancreatic acinar cells, which trigger the spiral of self-perpetuating tissue damage characteristic of acute pancreatitis [82], [83].

Interestingly, the anti-apoptotic factor Bcl-2/xL has been recently shown to inhibit PMCA activity in pancreatic acinar cells [78]. Although the pathophysiological relevance of this phenomenon remains to be determined, this observation highlights the critical importance of the PMCA in controlling cell fate, which may be very different depending on cell type or cellular context.

Paradoxically, the impairment of PMCA function and the subsequent dysregulation of cytosolic Ca^2+^ homeostasis can, in some cases, be cytoprotective [84]. During oxidative stress or tumour necrosis factor (TNF)-induced cell death, the accumulation and damage of lysosomes has been suggested to be important in determining cell fate [85], [86]. In TNF-resistant cell lines, in which PMCA4 is mutated, the resulting enhanced Ca^2+^ signalling has been shown to promote the exocytotic loss of lysosomes, resulting in protection against TNF-induced cell death [84]. This therefore suggests, somewhat counter-intuitively, that PMCA4 promotes TNF-induced cell death.

In MDA-MB-231 breast cancer cells the expression of different PMCA isoforms differentially regulate cell fate by protecting against either Ca^2+^-dependent necrosis (PMCA1) or caspase-dependent apoptosis (PMCA4) [19]. Specific siRNA-mediated knockdown of PMCA1 potentiated ionomycin-induced necrosis, whereas siRNA-mediated knockdown of PMCA4 potentiated Bcl-2 inhibitor (ABT-263)-mediated apoptosis and reduced NFkB nuclear translocation [19]. However, in colon cancer cells (HT-29), PMCA4 expression is downregulated, which appears to promote cell proliferation without any effect on the sensitivity of mitochondrial uncoupler (CCCP)-induced or TNFα-induced cell death pathways [87].

In breast epithelial cells, the PMCA has an important role in the normal physiology and in tumorigenesis. During lactation PMCA2 is up-regulated at the apical membrane where it contributes to apical Ca^2+^ transport and the majority of the Ca^2+^ composition within milk produced by normal mammary epithelial cells. During weaning, PMCA2 is down-regulated when the secretory cells die from apoptosis [88]. This loss of PMCA2 expression is thought to elevate resting Ca^2+^ thereby increasing the sensitivity of Ca^2+^-dependent apoptosis [89]. This is a form of “physiological” apoptosis known as mammary gland involution and remodelling of PMCA2 expression is central to this process. However, in breast cancer PMCA2 overexpression correlates with poor outcome due to resistance to apoptosis which is a major cancer hallmark [89].

Insulin-secreting β-cells (BRIN-BD11) also exhibit a functional paradox. Overexpression of PMCA reduces resting [Ca^2+^]_i_ and mitochondrial Ca^2+^ overload, which intuitively one might expect would protect against Ca^2+^-dependent apoptosis. However, this also leads to ER Ca^2+^ depletion, leading to ER-stress (activation of inositol-requiring enzyme 1α/X-box binding protein 1 pathway (IRE1α-XBP1) and inhibition of Activating Transcription Factor 6 (ATF6)) and the consequent activation of caspase-mediated apoptosis [90]. Therefore, in this context PMCA overexpression may induce apoptosis.

## 4 Metabolism, ATP supply and ATP dependency of PMCA

To better understand the paradoxical role of the PMCA in cell death, it is necessary to explore the mechanisms by which the PMCA is regulated, the ATP dependency and the supply of ATP to the PMCA. Intuitively, one might predict that ATP depletion would inhibit PMCA leading to Ca^2+^ overload and necrotic cell death [17], [18], [91], [92]. However, this is likely to be an over simplification because there are numerous factors that can influence the ATP sensitivity of the PMCA.

The PMCA has a high affinity catalytic ATP binding site (Km ∼ 3 μM) and lower affinity regulatory binding site (Km ∼ 145 μM) [93]. However, more recent studies suggest a more complex ATP dependency [94]. Most normal healthy cells have a resting ATP concentration in the mM range, suggesting that ATP has to drop 100–1000 fold before the PMCA is inhibited, which might only occur under severe prolonged metabolic stress?

Studies in HeLa cells showed that high, necrosis-inducing, ATP-depleting concentrations of H_2_O_2_ (32 mM) caused a Ca^2+^ overload response that was due to inhibition of the PMCA but that was also dependent on functional Na^+^-K^+^-ATPase activity [18]. The authors concluded that this was due to a thermodynamic shift of free energies of the pumps in favour of the Na^+^-K^+^-ATPase [18]. It was argued that he H_2_O_2_ also induced a Na^+^ influx that reduced the free energy for the Na^+^ pump, due to the dissipation of the Na^+^ gradient, without any appreciable effect on the free energy for the PMCA (Ca^2+^ gradient maintained). Therefore, ATP reaches a critical concentration where it becomes more energetically favourable to pump Na^+^ than Ca^2+^ and so the PMCA is inhibited in favour of a fully functional Na^+^-K^+^-ATPase. In other words the Na^+^-pump “steals” ATP from the PMCA [18]. This study suggests that the PMCA may be inhibited by sub-maximal ATP depletion

The extent of ATP depletion will largely depend on whether mitochondria or glycolysis is inhibited, whether ATP is being rapidly consumed and the extent of PMCA inhibition will depend on whether there is a localised ATP supply. Moreover, would inhibition of mitochondrial metabolism alone deplete ATP levels sufficiently to inhibit the PMCA, especially if glycolytic ATP production remains active? The classic textbook view is that mitochondria are much more energetically efficient than glycolysis in metabolising glucose. For example, mitochondrial oxidative phosphorylation produces 32 molecules of ATP, whereas glycolysis produces only 2 molecules of ATP for every glucose molecule metabolised. However, this is likely to be an over simplification as most cells exhibit metabolic plasticity and are able to adapt to their environment (i.e. as observed in hypoxic tissues). Cancer cells are an extreme example of this and often undergo a metabolic switch from mitochondrial metabolism to glycolysis, due to mutations within key mitochondrial enzymes and the up-regulation of glycolytic enzymes [95], [96]. This phenomenon is known as the Warburg effect, named after Otto Warburg, who first described the increase in glycolysis in cancer cells in the 1920s [97]. Our recent studies in pancreatic cancer cell lines were the first to show that the PMCA is critically reliant on glycolytically-derived ATP [14], [15]. Specifically, inhibition of glycolysis, but not mitochondrial metabolism, resulted in ATP depletion, PMCA inhibition, cytotoxic Ca^2+^ overload and ultimately necrotic cell death [98]. This was reversed when cells were cultured in glucose-free media supplemented with either galactose or ketoisocaproate to reduce cells’ reliance on upregulated glycolytic rate [15]. This suggests that in highly glycolytic pancreatic cancer cells exhibiting the Warburg effect, glycolytically-derived ATP, rather than mitochondrially-derived ATP is more important for maintaining PMCA function and cell survival.

These observations are particularly important when one considers that the PMCA is reported to have its own local, sub-membrane supply of glycolytically-derived ATP that may render it largely insensitive to inhibition of mitochondrial metabolism [99], [100], [101] (Fig. 3). Specifically, in isolated inside-out plasma membrane vesicles from pig stomach smooth muscle enriched with PMCA, an endogenous membrane-bound glycolytic system was observed which provided ATP to fuel PMCA-dependent Ca^2+^ uptake [99], [100]. Moreover, providing glycolytic *substrates* were present, the Ca^2+^ uptake (PMCA activity) persisted in the absence of an exogenously applied ATP regenerating system [99], [100]. In addition, studies in human erythrocytes which lack mitochondria have shown that several glycolytic enzymes associate with the plasma membrane, either via band 3 protein (anion-exchanger-1) [102], [103], [104], [105] or via phospholipids [106]. Moreover, the PMCA has been shown to reside within caveolae, where these phospholipids are enriched and regulate the activity of the PMCA [107]. Finally, it has been suggested that a localised pool of ATP, associated with the cytoskeleton, provides a privileged ATP supply to the PMCA [108]. Together these data implicate the possibility that a localised glycolytic ATP supply to PMCA may independently regulate PMCA activity regardless of whether global cellular ATP is maintained.

In most healthy cells under physiological conditions, when the bulk cytosolic ATP concentration is saturating for the PMCA (i.e.> 1 mM), such close functional coupling between glycolytic enzymes and the PMCA is likely to be of minimal functional significance. In other words, the PMCA does not care whether ATP comes from a mitochondrial or glycolytic source. However, in the face of impaired mitochondrial metabolism, for example under conditions of cellular stress, a glycolytic source of ATP might be critical for maintaining PMCA activity and thus restoring low resting cytosolic [Ca^2+^]_i_. Indeed, acute metabolic stress induced by pancreatitis-inducing agents markedly inhibited PMCA in pancreatic acinar cells [16], [17], [109], [110], which was attenuated by treatment with insulin [16], [17]. This insulin protection was due to an acute “cancer-like” switch from mitochondrial metabolism to glycolysis which was sufficient to preserve the ATP supply to the PMCA, thereby preventing cytotoxic Ca^2+^ overload and necrotic cell death [16], [17]. Under these stressed conditions, a “privileged” local glycolytic ATP supply (or more specifically an insulin-mediated “up-regulated” glycolytic supply of ATP) may be sufficient to “fuel” the PMCA, even if bulk (global) ATP is close to zero. Conversely, it is also possible that inhibition of such a localised ATP supply to the PMCA might inhibit the PMCA even when global ATP is maintained, which might be sufficient to activate Ca^2+^-dependent apoptosis but not necrosis.

### 4.1 Regulation of the PMCA by acidic phospholipids

Another important caveat when considering the ATP-sensitivity of the PMCA is that acidic phosphoplipids, such as phosphatidylinositol (PI), phosphatidylcholine (PC) and phosphatidylserine (PS) regulate the ATP sensitivity of the PMCA and mimic regulation by CaM [23], [24]. Loss of PS (or PI) from the lipid environment of the PMCA lowered the affinity of the PMCA for ATP (Kd, 5–10 mM, regulatory site) [111], [112], suggesting that disruption of the lipid environment around the PMCA may be sufficient to render the PMCA highly sensitive to ATP depletion. However, this evidence is based on *in vitro* cell-free assays of ATPase activity, whereby PS/PI was either absent or present in an artificial membrane, making it difficult to extrapolate these findings to intact cells. It is therefore unclear what the critical concentration of PS is to maintain “normal” ATP-sensitivity of the PMCA or whether this relationship is influenced by dynamic changes in Ca^2+^, Mg^2+^, CaM or other membrane lipids. However, functional studies in intact endothelial cells have shown that the loss of phosphatidylserine from the inner leaflet of the plasma membrane, following cholesterol depletion with β-methyl-cyclodextrin, inhibited PMCA activity [107]. This has important implications for apoptosis, since PS is known to line the inner leaflet of the plasma membrane and a proportion is thought to flip to the extracellular side of the membrane during apoptosis [113]. This provides the dying cell with an “eat me” signal detected by macrophages that then phagocytose the dying cell from the tissue [113]. Furthermore, the enzyme responsible for this PS assymetry within the plasma membrane (aminophospholipid translocase or flippase) [114] requires millimolar ATP [115], [116] and is inhibited by oxidative stress [117], [118]. Collectively these studies suggest that cellular stress may have a profound effect on the ATP sensitivity of the PMCA, and thus inhibition of the PMCA might be observed even with only mild ATP depletion. Moreover, this altered ATP sensitivity of the PMCA during apoptosis might provide a feedforward potentiation of Ca^2+^-dependent apoptosis before ATP declines sufficiently to trigger necrosis.

### 4.2 Effect of mitochondrial-derived reactive oxygen species

Severe mitochondrial stress, whatever the mechanism, often leads to the generation of reactive oxygen species (ROS) [119]. Furthermore, there is also good evidence that oxidants (H_2_O_2_) can directly oxidise PMCA and also oxidise calmodulin, which is the main activator of PMCA [120]. Hence, metabolically derived ROS may have a profound inhibitory effect on PMCA activity. In addition, H_2_O_2_ has been reported to reduce the functional expression of PMCA at the plasma membrane of cultured hippocampal neurons within 1–2 h [121]. Such rapid changes in functional expression of PMCA at the plasma membrane could lead to reduced Ca^2+^ efflux during metabolic stress even in the presence of continued high ATP levels.

### 4.3 Calpain/caspase cleavage of the PMCA

The release of cytochrome C from the mitochondria and the subsequent activation of caspases and calpain [122] have both been reported to cleave and eventually lead to the inactivation of the PMCA [20], [123], [124]. It is interesting to note that the time-frame over which cytochrome C release can occur (>2 mins) [125] coincides with the time the PMCA can be observed to be inhibited, and happens well before ATP depletion was observed [109].In fact the initial cleavage by caspase and calpain actually activates the pump, but through the subsequent internalisation and degradation of the PMCA the protease effect is manifested as inhibition of the pump [126], [127]. Interestingly, specific caspase-3 cleavage of PMCA4b produces a 120 kD fragment that is constitutively activated, due to the removal of the autoinhibitory domain [72], [73], [74]. It is also interesting to note that calpain can also be directly activated by H_2_O_2_ and Ca^2+^ [128].

## 5 Summary

In summary the PMCA represents an important regulator of cell death, both apoptosis and necrosis. Although impaired PMCA activity is generally regarded as being cytotoxic and the maintenance of PMCA activity is regarded as being cytoprotective, this rather simplistic view is by no means clear-cut. The PMCA can be differentially regulated by a vast repertoire of additional signalling pathways, proteolytic cleavage and the ATP-dependency and ATP supply is even more complex than originally thought. This raises the question as to whether the PMCA deserves greater recognition in the Pantheon of calcium signalling machinery than it currently has. Moreover, the regulation of the PMCA may offer a relatively untapped and rich tapestry of novel therapeutic targets for a variety of diseases in which Ca^2+^-dependent cell death is central.
